# Transformational Leadership, Psychological Safety, and Concussion Reporting Intentions in Team-Sport Athletes

**DOI:** 10.3390/ijerph22030393

**Published:** 2025-03-07

**Authors:** John Batten, Matthew J. Smith, Janet Young, Abi Braim, Rebecca Jull, Callum Samuels, Alan J. Pearce, Adam J. White

**Affiliations:** 1 Department of Sport, Allied Health and Social Work, University of Winchester, Hampshire SO22 4NR, UK; matt.smith@winchester.ac.uk (M.J.S.); a.braim.18@unimail.winchester.ac.uk (A.B.); r.jull.18@unimail.winchester.ac.uk (R.J.); c.samuels.18@unimail.winchester.ac.uk (C.S.); 2 First Year College, Victoria University, Footscray Campus, Melbourne, VIC 8001, Australia; janet.young@vu.edu.au; 3 School of Health Science, Swinburne University of Technology, Melbourne, VIC 3122, Australia; 4 The Professional Footballers’ Association, Lincoln Building, Lincoln Square, Manchester M2 5AD, UK; adam.white@thepfa.com

**Keywords:** concussion reporting, mild traumatic brain injury, psychological safety, transformational leadership

## Abstract

**Background/Objectives:** The present study examined the predictive relationships between transformational leadership, psychological safety, and concussion reporting intentions. Interviews were used to understand the impact of the coach and teammates on the team environment and how this might lead to greater concussion reporting in team-sport athletes. **Methods:** This study employed a mixed-methods explanatory sequential design. 233 team-sport athletes (*n* = 160 males, *n* = 73 females, *mean age* = 19.83 years, *SD* = 3.15) completed quantitative measures of transformational leadership, psychological safety, and concussion reporting intentions, while five participants (*n* = 2 males, *n* = 3 females, *mean age* = 18.40 years, *SD* = 0.55) were subsequently interviewed about their experiences. **Results:** Quantitative results indicated that transformational leadership predicted a psychologically safe environment (*p* < 0.001), and social norms for team-sport athletes predicted concussion reporting intentions (*p* < 0.001). Qualitative findings highlighted the important role that coaches and teammates play in creating an open environment that encourages concussion reporting. Education and awareness were also seen to further develop such an environment, while athletes identified several barriers to reporting symptoms of concussion. **Conclusions:** The findings from this study provide researchers and practitioners with preliminary evidence that can shape the design of interventions to support the development of a team environment that can promote concussion reporting and, thus, prioritise player health.

## 1. Introduction

Concussion is a form of mild traumatic brain injury [1], caused by either a direct force to the head or indirectly when an impulsive force to the neck or upper body is transferred to the head [2,3]. A concussion may, but often does not, involve loss of consciousness [4], making it a complicated injury that does not always present with obvious signs or symptoms [5]. Nevertheless, concussion is commonplace in contact sports [6,7], with emerging evidence suggesting that repeated (concussive and non-concussive/non-clinical) impacts to the head may lead to increased risk of cognitive and mental health issues and/or movement disorders in later life [8,9,10,11], as well as associated deterioration in brain tissue [12]. Although a missed concussion diagnosis might lead to a subsequent increase in short- and/or long-term injury for affected sportspeople [13,14], many athletes do not report symptoms of concussion [15,16,17,18,19], which has implications for player health, wellbeing, and performance.

Various reasons might explain why athletes do not report symptoms of concussion for themselves or for their teammates. These include a perception that symptoms are not serious enough to report, not wanting to be removed from play, as well as not wanting to ‘let down’ teammates or coaches [20,21]. Unfortunately, while education about the risks of concussion is important, education alone is unlikely to motivate athletes to report symptoms of concussion [22]. Thus, to facilitate reporting, interventions have been developed to change the culture of a team in respect to concussion. One such intervention is the ‘Team Up Against Concussion’ programme developed by the Concussion Legacy Foundation in the United States. Although there is currently limited evidence to support the efficacy of such programmes, athletes have been found to be more likely to encourage a teammate to seek help when there is a perceived negative impact on either a player’s health or their on-pitch performance [23].

Milroy et al. [24] emphasise how multiple factors can impact concussion reporting. As such, researchers and practitioners should use theory to guide investigations into concussion reporting to better understand where and how to intervene. One such theory that has been used to support investigations into concussion reporting is the Theory of Reasoned Action and Planned Behaviour (TRA/TPB) [25]. This theory contends that attitudes toward a behaviour (a person’s overall assessment of the behaviour), subjective or social norms surrounding a behaviour (how much social pressure a person feels to perform or not perform the behaviour), as well as perceived control over a behaviour (how much control an individual feels they have over the behaviour) are associated with intentions to perform a behaviour [25,26]. The current study aims to build on previous research that has considered the TRA/TPB in relation to concussion reporting by examining factors that may impact individual attitudes, subjective norms, and perceived behavioural control. One such factor is the team environment and, specifically, levels of psychological safety within the team.

Psychological safety is a ‘shared belief held by members of a team that the team is safe for interpersonal risk taking’ (p. 350) [27], such as asking for help, or seeking feedback from others. Within a psychologically safe environment, team members are genuinely interested in their teammates, have positive intentions towards one another, and feel valued and appreciated by their peers [28,29]. Psychologically safe environments also alleviate concerns about how others will react, supporting risk taking without fear of embarrassment or rejection [27,29]. In contrast, when members of a team feel psychologically unsafe, they are less likely to show vulnerabilities that threaten their self-image; due to concerns about appearing weak [27]. Thus, it was hypothesised that psychologically safe environments will result in concerns about letting down teammates or coaches [21] being alleviated, with social norms that support concussion reporting and prioritise player health [23,30]. Further, a team culture that includes improvements in information sharing [28,31] should increase perceptions of control over reporting symptoms of concussion.

The current study also aims to examine how to nurture psychologically safe environments in sport. Here, previous research from business and organisational settings, e.g., [32,33,34,35,36], suggests that transformational leadership is key in the development of psychological safety. Indeed, existing literature [36,37] suggests that transformational leaders might support the development of a psychologically safe environment in four main ways. First, using idealised influence, transformational leaders may be able to develop psychologically safe environments by treating members equally and focusing on co-operation as opposed to competition between members. Second, transformational leaders might use intellectual stimulation to inspire members to take interpersonal risks, challenge social norms, and view problems from different perspectives. Third, individualised consideration may encourage psychological safety when transformational leaders attend to the needs of individual members and encourage open and honest communication. Finally, transformational leaders that show inspirational motivation might support the development of psychologically safe environments through the creation of shared values and a collaborative approach.

**Figure 1:**
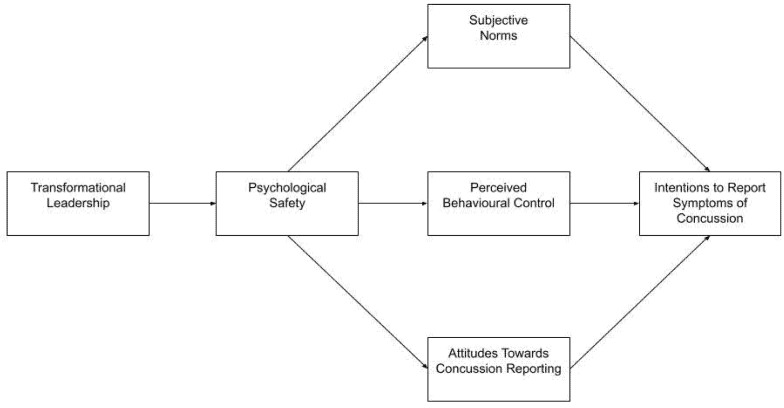
Hypothesised relationships between transformational leadership, psychological safety, subjective norms, attitudes, perceived behavioural control, and intentions to report symptoms of concussion.

Overall, the present study builds on existing literature in several ways. First, the present study extends on the literature examining concussion reporting intentions (e.g., [16,17,19,20,21]), and the limited literature examining the relationship between transformational leadership and psychological safety in sport [38], through examining the relationships between transformational leadership, psychological safety, and concussion reporting intentions in team-sport athletes. Here, it was first hypothesised that transformational leadership would significantly predict psychological safety (Hypothesis 1); second, that psychological safety would significantly predict perceived behavioural control, subjective norms, and individuals’ attitudes towards reporting their own symptoms of concussion (Hypothesis 2a) *and* symptoms of concussion in their teammates (Hypothesis 2b); and third, that perceived behavioural control, subjective norms, and attitudes would significantly predict intentions of individuals to report their own symptoms of concussion (Hypothesis 3a) *and* symptoms of concussion in their teammates (Hypothesis 3b) (see Figure 1).

Furthermore, Stentz et al. [39] suggests that research examining the relationship between transformational leadership and psychological safety in sport would benefit from a multi-method approach. Thus, the present study extends on previous literature by using qualitative methods to help explain the complex interplay between coaches as transformational leaders, psychological safety, and concussion reporting intentions in team-sport athletes. From an applied perspective, while other factors (e.g., education) have a role to play, the development of a psychologically safe environment could become an important consideration for interventions that seek to enhance concussion reporting in team-sport settings. Thus, a further aim of the current study was to provide researchers and practitioners with preliminary evidence to help shape the development of interventions that can improve concussion reporting and, in turn, enhance the health and wellbeing of athletes.

## 2. Materials and Methods

### 2.1. Study Design

This study adopted a pragmatic research philosophy [40], underpinned by methodological pluralism (drawing on both positivist and interpretivist philosophies) as opposed to methodological puritanism (use of a single paradigm). Specifically, a mixed-methods explanatory sequential study design [41] was employed. Here, quantitative data were used to examine the relationships between transformational leadership, psychological safety, and concussion reporting intentions, while qualitative methods were used to further understand the impact of the coach and teammates on the team environment, as well as concussion reporting intentions and behaviours.

### 2.2. Participants

Quantitative data were collected from 233 student–athletes (*n* = 160 males, *n* = 73 females) from two Australian universities. The mean age of student–athletes was 20 years (*M* = 19.83 years, *SD* = 3.15). All student–athletes were competing at a recreational level (local, formal competition) from across a variety of team-sports, including Australian rules football (*n* = 87), association football/soccer (*n* = 48), basketball (*n* = 37), netball (*n* = 27), cricket (*n* = 18), volleyball (*n* = 7), American football (*n* = 3), field hockey (*n* = 2), rugby league (*n* = 2), baseball (*n* = 1), and softball (*n* = 1). The student–athletes had spent a mean of four years with their current team (*M* = 4.22 years, *SD* = 3.61) and a mean of two years with their coach (*M* = 2.01 years, *SD* = 1.95).

Qualitative data were collected from five student–athletes (*n* = 2 males, *n* = 3 females). The mean age of these student–athletes was 18 years (*M* = 18.40 years, *SD* = 0.55). All participants had competed in their team-sport (*n* = 2 association football/soccer, *n* = 1 Australian rules football, *n* = 1 basketball, *n* = 1 volleyball) at a recreational level (local, formal competition) for a minimum of two years (*M* = 5.60 years, *SD* = 2.79). These inclusion criteria were chosen to ensure that participants could provide in-depth and rich detail about their experiences of coaching and leadership, team environments, as well as concussion reporting intentions and behaviours.

### 2.3. Procedure

Student–athletes were invited to participate in an anonymous survey following general announcements across taught classes. The survey included information about the study aims, requirements, and expectations for participation, as well as an informed consent form. Student–athletes completed the survey using JISC online surveys. In addition to the online survey, five student–athletes provided informed consent to be interviewed. These student–athletes were offered various time slots (accounting for time zone differences) before a mutually convenient time and date was agreed for each interview. All interviews were conducted and recorded using Microsoft Teams. Participants were reminded that they did not have to answer any questions if they did not wish to do so and that they could stop the interview at any point and/or withdraw their involvement in the study without penalty. No incentives were provided to participants, and the study was approved by the institutional ethics committees of authors one and three (HWB_REC_21_01_Smith and HRE16-237).

### 2.4. Quantitative Measures

#### 2.4.1. Transformational Leadership

To assess perceptions of transformational leadership in coaches, participants completed the *Differentiated Transformational Leadership Inventory* [42]. This inventory consists of 27 items and seven sub-scales, reflecting various transformational leadership behaviours. Each item from the inventory is scored on a 5-point Likert scale ranging from 1 (not at all) to 5 (all the time). However, Exploratory Factor Analysis (EFA) revealed that several items cross-loaded or misloaded from an intended factor (sub-scale). As such, data from all 27 items were collated into one transformational leadership factor, explaining approximately 46% of the cumulative variance, with this single-factor solution used in all analyses. The Cronbach’s alpha reliability coefficient [0.952] for this factor was >0.70, indicating adequate internal reliability [43].

#### 2.4.2. Psychological Safety

Participants completed the *Team Psychological Safety* questionnaire [27] to assess perceptions of psychological safety within their team. This questionnaire included 7 items (e.g., It is safe to take a risk in this team) and required participants to rate their level of agreement with each item on a 7-point scale ranging from 1 (strongly disagree) to 7 (strongly agree). Here, higher scores indicated a greater perceived sense of psychological safety within the team. EFA found all items to load onto one factor, explaining approximately 39% of the cumulative variance. In addition, the Cronbach’s alpha reliability coefficient [0.722] for this questionnaire was >0.70, which was deemed to be acceptable [43].

#### 2.4.3. Concussion Reporting Intentions

The *Concussion Reporting Intentions Scale (CRIS)* used in this study included eight items. Two of these items assessed attitudes towards concussion reporting, with higher scores indicating a more favourable attitude towards reporting symptoms of concussion. Two items assessed subjective norms, with higher scores indicating that social referents feel more positive towards the reporting of symptoms of concussion. Two further items assessed perceived behavioural control, with higher scores indicating more feelings of control over concussion symptom reporting, while the final two items assessed intentions to report symptoms of concussion.

When completing the CRIS, participants were provided with a definition of concussion against which they could respond to items related to symptom reporting. This definition stated that ‘A concussion is an injury caused by a blow to the head or sudden movement of the body followed by a variety of signs and symptoms that may include any of the following: headache, dizziness, loss of balance, blurred vision, ‘seeing stars’, feeling in a fog, or slowed down, memory problems, poor concentration, nausea, or throwing up. Getting ‘knocked out’ or being unconscious does *not* always occur with a concussion’ [44].

The first CRIS used in this study examined the willingness of individuals to report their own symptoms of concussion. All items started with the same ‘anchor’, which stated ‘When I experience possible symptoms of concussion…’. The second CRIS used in this study examined the willingness of individuals to report possible symptoms of concussion in their teammates. Although the ‘anchor’ was again the same for all items, it was changed to ‘When I see a teammate displaying possible symptoms of concussion…’. Participants responded to each item on a 7-point Likert scale, ranging from 1 (very strongly disagree) to 7 (very strongly agree). Measures of attitudes, subjective norms, perceived behavioural control, and intentions to report symptoms of concussion were calculated by summing items for each sub-scale.

EFA supported a four-factor model that represented the willingness of individuals to report their own symptoms of concussion, explaining approximately 88% of the cumulative variance. Cronbach’s alpha reliability coefficients for all sub-scales were also >0.70, indicating adequate internal reliability [43]. Similarly, a second EFA supported a four-factor model that represented the willingness of individuals to report symptoms of concussion in their teammates, explaining approximately 88% of the cumulative variance. Again, Cronbach’s alpha reliability coefficients for all sub-scales were >0.70. More information about the development of the CRIS can be found in Supplementary File S1.

### 2.5. Qualitative Data Collection

An interview guide was developed following a five-stage process [45]. This guide provided consistency while allowing for flexibility and expansion through probes (where necessary) to support more detailed responses from participants [46]. The first stage informed participants of the purpose of the interview. The second stage outlined research ethics, including gaining informed consent from participants. The third stage focused on building rapport with participants, such as asking open questions about their sport. The fourth stage was divided into different sections (e.g., leadership, psychological safety, concussion reporting), with questions informed by the literature in the area, as well as the results from the quantitative analyses. The final stage involved asking participants if there was anything else they would like to add or ask before debriefing them and concluding the interview by thanking them for their time and participation.

As part of the interview process, vignettes were also used to allow researchers to explore sensitive topics without bringing harm or embarrassment to participants [47]. The vignettes used in this study were developed from newspaper articles examining concussion in sport and verbally relayed to participants. The use of ‘real-life’ examples (as opposed to fictitious accounts) gave the participants something they could relate to [48], as well as reminding them that they are not alone in their experiences. The use of vignettes also allowed participants to control how much they shared about their personal experiences [49], supporting their limits and comfort within the interview. An example vignette used in this study was as follows:

*Shona McCallin [international field hockey player and Olympic gold medallist] said that when she got a concussion in 2018, she was angry about being taken out of the game because she believed she was fine. However, later when she went to warm down with her teammates, she couldn’t run or co-ordinate herself and found it hard to talk.*

This vignette was followed by questions such as, ‘How do coaches in your team manage someone displaying possible concussive symptoms like Shona McCallin?’ and ‘What would you do if you saw a teammate displaying symptoms like this’?

### 2.6. Data Analysis

#### 2.6.1. Analysis of Quantitative Data

Analyses of regression using the enter method were conducted to determine the extent to which independent variables significantly predicted dependent variables. First, analysis of regression was used to determine the extent to which transformational leadership predicted psychological safety (Hypothesis 1). Second, analyses of regression were used to determine the extent to which psychological safety predicted perceived behavioural control, subjective norms, and individuals’ attitudes towards reporting their own symptoms of concussion (Hypothesis 2a) *and* symptoms of concussion in their teammates (Hypothesis 2b). Third, analyses of regression were used to determine the extent to which perceived behavioural control, subjective norms, and attitudes predicted individuals reporting their own symptoms of concussion (Hypothesis 3a) *and* symptoms of concussion in their teammates (Hypothesis 3b). The alpha level was set at 0.05 for all analyses. The relative strength of the predictions of independent variables (i.e., standardised beta coefficients) were interpreted using the recommendations of Cohen [50], who defined values near 0.02 as small, near 0.15 as medium, and above 0.35 as large. All analyses were undertaken using the Statistical Package for Social Sciences (SPSS) Version 27.

#### 2.6.2. Analysis of Qualitative Data

Interviews lasted between 44 and 82 min (*M* = 64.67 min) and were all transcribed verbatim using Microsoft Teams. A thematic analysis was then undertaken, which involved organising and summarising data in relation to the research question [51]. Here, researchers immersed themselves in data by listening to the recordings, reading through the transcripts, highlighting, and then grouping key words and phrases into meaningful codes. During the coding process, common themes were identified and noted down, before being discussed and reviewed once completed. Thematic maps were also produced with the aim of developing an organised category system highlighting the impact of the coach and teammates on the team environment, as well as concussion reporting intentions and behaviours. To ensure a rigorous qualitative process, critical friends were used to challenge ideas, consider different interpretations, as well as encourage reflection and discussion [52]. For instance, critical friends challenged themes and codes for relevance, ensuring that clear rationales were developed for each theme and code therein. Reflexive memos were also made after each interview to help identify any personal values or opinions that could impact the research [53]. For instance, one participant played a sport at a similar level to one of the researchers, with the reflexive memo noting how effort was made not to make assumptions when asking questions.

## 3. Results

### 3.1. Quantitative Results

Descriptive statistics that consider perceptions of transformational leadership, psychological safety, and concussion reporting intentions for male and female team-sport athletes are presented in Supplementary File S2, while tables that include all analyses of regression, as well as confidence intervals, standardised beta coefficients, significance, and *R*^2^ adjusted values can be found in Supplementary File S3.

In line with Hypothesis 1, analysis of regression indicated a significant (*p* < 0.001), positive, large (β = 0.469) relationship between transformational leadership and psychological safety within teams, explaining 22% of the variance.

With regards to reporting their own symptoms of concussion, analysis of regression for Hypothesis 2a revealed a significant (*p* < 0.001), positive, large (β = 0.384) relationship between perceptions of psychological safety within teams and perceived behavioural control, explaining 15% of the variance. Further, analysis of regression indicated a significant (*p* < 0.001), positive, large (β = 0.380) relationship between perceptions of psychological safety within teams and subjective norms, explaining 14% of the variance. In addition, analysis of regression revealed a significant (*p* < 0.001), positive, large (β = 0.453) relationship between perceptions of psychological safety within teams and attitudes towards concussion reporting, explaining 20% of the variance.

When reporting symptoms of concussion in teammates, analysis of regression for Hypothesis 2b indicated a significant (*p* = 0.001), positive, medium (β = 0.219) relationship between perceptions of psychological safety within teams and perceived behavioural control, explaining 4% of the variance. Analysis of regression also indicated a significant (*p* = 0.024), positive, small (β = 0.148) relationship between perceptions of psychological safety within teams and subjective norms, explaining 2% of the variance. In addition, analysis of regression revealed a significant (*p* < 0.001), positive, large (β = 0.356) relationship between perceptions of psychological safety within teams and attitudes towards concussion reporting, explaining 12% of the variance.

In terms of intentions to report symptoms of concussion, analysis of regression for Hypothesis 3a revealed positive relationships between perceived behavioural control, subjective norms, and attitudes of individuals to report their own symptoms of concussion, explaining 36% of the variance. Here, a non-significant (*p* = 0.536) relationship between perceived behavioural control and concussion reporting intentions was observed. In contrast, a significant (*p* < 0.001), large (β = 0.501) relationship between subjective norms and concussion reporting intentions was shown. In addition, a significant (*p* = 0.038), small (β = 0.144) relationship between attitudes and concussion reporting intentions was observed.

With regards to reporting symptoms of concussion in their teammates, analysis of regression for Hypothesis 3b revealed positive relationships between perceived behavioural control, subjective norms, and attitudes of individuals towards concussion reporting, explaining 47% of the variance. Here, a non-significant (*p* = 0.334) relationship between perceived behavioural control and concussion reporting intentions was observed. Conversely, a significant (*p* < 0.001), large (β = 0.590) relationship between subjective norms and concussion reporting intentions was shown. Finally, a non-significant (*p* = 0.065) relationship between attitudes and concussion reporting intentions was observed.

### 3.2. Qualitative Findings

**Figure 2:**
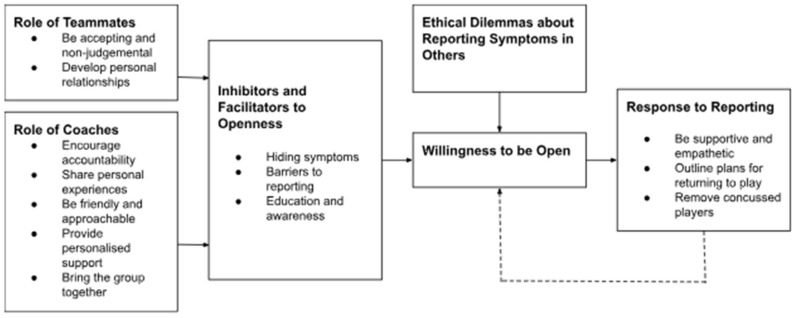
Factors influencing concussion reporting intentions in team-sport athletes.

Following thematic analysis, six main themes were identified: (1) role of teammates; (2) role of coaches; (3) inhibitors and facilitators to openness; (4) open team environment; (5) ethical dilemmas about reporting symptoms in others; and (6) response to reporting; which impacted concussion reporting intentions. These main themes (in bold) and associated sub-themes are displayed in Figure 2.

Data presented in Table 1 illustrates what teammates and coaches do to support the development of an open team environment (see Main Themes 1 and 2). In addition to the role of teammates and coaches, data highlight a range of inhibitors and facilitators to being open about discussing concussion (see Main Theme 3), with education and awareness one factor that might develop such an open environment.

Collectively, these factors impact the openness of the team environment in relation to concussion reporting (see Main Theme 4). However, ethical dilemmas about reporting symptoms of concussion in teammates who may have confided in them were also presented (see Main Theme 5).

When athletes do report symptoms of concussion to others, the response of others to reporting also appeared to influence the openness of the team environment (see Main Theme 6). For example, teammates were reported to be generally supportive and empathetic, with a focus on player welfare. Yet, how a coach reacts to one concussion appeared to impact the willingness of players to be open about similar injuries in the future. Here, the importance of removing concussed players, having clear plans in place about returning to play, as well as providing reassurance to players, were considered key.

## 4. Discussion

The present study examined the relationships between transformational leadership, psychological safety, and concussion reporting intentions in team-sport athletes. In line with the hypotheses, quantitative results indicated that perceptions of transformational leadership in coaches significantly predicted perceptions of psychological safety within teams (Hypothesis 1); that perceptions of psychological safety within teams significantly predicted perceived behavioural control, subjective norms, and individuals’ attitudes towards reporting their own symptoms of concussion (Hypothesis 2a) *and* symptoms of concussion in their teammates (Hypothesis 2b); and that subjective norms and attitudes significantly predicted intentions of individuals to report their own symptoms of concussion (Hypothesis 3a) *and* that subjective norms significantly predicted intentions of individuals to report symptoms of concussion in their teammates (Hypothesis 3b). In other words, transformational leaders were found to support the development of a psychologically safe environment, within which social norms encouraged team-sport athletes to report their own symptoms of concussion and symptoms of concussion in their teammates.

Considering Hypothesis 1, transformational leaders might support the development of a psychologically safe environment in four main ways: using idealised influence, intellectual stimulation, individualised consideration, and inspirational motivation [35]. However, the qualitative results from the present study suggest that individualised consideration may be particularly important for the development of a psychologically safe environment amongst team-sport athletes. Indeed, individualised consideration might promote psychological safety when transformational leaders attend to the needs of individual members and encourage open and honest communication. Specifically, participants in the present study expressed a desire for a coach who is friendly, approachable, and open to talking about both sport-specific and personal issues with players (see Main Theme 2). Further, participants discussed the importance of coaches giving players individual attention, showing genuine interest in them, as well as displaying empathy towards players. Resultantly, coaches who engage in such behaviours might be able to create an open—and arguably psychologically safe—environment that involves regular communication between players and coaches, including discussions about concussion.

Although this study sought to examine the role of coaches in developing a psychologically safe environment, qualitative data suggested that teammates also play an important role in creating such an environment in sport settings (see Main Theme 1). This finding is in line with previous research [54] that has investigated the role of athlete leaders in influencing team culture. Specifically, participants in this study highlighted a need for their teammates to be open, accepting, and non-judgemental. Participants also spoke about how cultivating personal friendships helps players to feel more comfortable with their teammates, leading to the development of a supportive environment. Here, participants discussed the importance of showing interest in teammates and how talking about things other than sport helps to improve relationships with them. Further, participants noted how spending time together away from sport helps to improve relationships with teammates. Such findings support previous research (e.g., [28,29]) that found team members to support the development of a psychologically safe environment by showing genuine interest in their teammates, having positive intentions towards one another, and valuing and appreciating their peers.

Considering the Theory of Reasoned Action and Planned Behaviour, this study examined the extent to which psychological safety within teams predicted perceived behavioural control, subjective norms, and individuals’ attitudes towards reporting their own symptoms of concussion *and* symptoms of concussion in their teammates. In line with Hypotheses 2a and 2b, quantitative results indicated that attitudes towards concussion reporting improved when perceptions of psychological safety increased. Similarly, when psychological safety increased, subjective norms and perceptions of control over reporting symptoms of concussion increased. These findings were consistent when participants considered reporting their own symptoms of concussion *and* symptoms of concussion in their teammates. Although the present study is the first to examine the link between psychological safety and concussion reporting intentions in sport, findings from previous research might help us to explain these observations. For instance, within a psychologically safe environment, concerns about letting down teammates or coaches might be alleviated with social norms that support concussion reporting and prioritise player health [23,30]. In addition, a team culture that includes improvements in information sharing [28,31] might increase how much control an individual feels they have over reporting symptoms of concussion.

Within a psychologically safe environment, quantitative results from this study found partial support for Hypotheses 3a and 3b. Specifically, results indicated that subjective norms and attitudes significantly predicted intentions of individuals to report their own symptoms of concussion (Hypothesis 3a) *and* that subjective norms significantly predicted intentions of individuals to report symptoms of concussion in their teammates (Hypothesis 3b). Alongside these quantitative findings, examination of qualitative data identifies the important role that subjective norms may play in encouraging team-sport athletes to report their own symptoms of concussion and symptoms of concussion in their teammates. Here, qualitative data from the present study suggested that regular communication between players and coaches that includes discussions about concussion helps to set clear expectations about what to do when concussions occur, including who players can speak to about such issues (see Main Theme 4). In turn, these expectations may serve to increase the social pressure experienced by team-sport athletes to report their own symptoms of concussion and symptoms of concussion in others. While attitudes were found to predict intentions of individuals to report their own symptoms of concussion, social norms were found to predict intentions of individuals to report their own symptoms of concussion *and* symptoms of concussion in their teammates. Thus, interventions that target social norms surrounding concussion reporting may be key in improving intentions to perform this behaviour [25,26,55].

One intervention that seeks to change the culture of a team in respect to concussion is the ‘Team Up Against Concussion’ programme developed by the Concussion Legacy Foundation in the United States. Although the present study did not evaluate the efficacy of this programme, the findings from this study suggest that similar interventions that develop transformational leaders, psychological safety, and subjective norms around reporting symptoms of concussion should be encouraged. Such interventions should focus on the needs of individual members and encourage regular, open, and honest communication about both sport-specific and personal issues. These interventions should target individual players, teammates, and coaches, with a focus on cultivating open, accepting, empathetic, non-judgemental, and supportive environments, within which discussions about concussion can take place. At the same time, interventions should set clear expectations about what should happen when concussions occur, including who players can speak to about such issues. In so doing, researchers and practitioners may be able to support the development of a team culture that promotes concussion reporting and prioritises player health [23,30].

Alongside interventions that target team culture, qualitative data from the present study identified a need for more education about concussion, as well as public discussions and role models from within sport, with players talking openly about their experiences (see Main Theme 3). While beyond the scope of this study, increased knowledge and attitudes regarding the consequences of concussion has been found to improve adherence to post-concussive return-to-play protocols [56]. Further, concussion education programmes have been found to improve the injury-preventing behaviours of players [57]. In terms of public discussions, participants noted how conversations about sport-specific concussion guidelines can help players to identify problematic behaviours in their own teams. Thus, while there are often various sociocultural discourses and misconceptions regarding concussion [58], how the media present and frame such discussions could support concussion reporting behaviours in sport. Similarly, former professional athletes talking about their experiences living with cognitive and mental health issues and/or movement disorders in later life has served to raise awareness about the risks associated with repeated impacts to the head from playing team sports [8,9,10,12] Thus, future researchers and practitioners must consider a multifactorial approach to concussion education and prevention [20,55].

Interventions, education, and improved public awareness about concussion in sport might also help to challenge potential inhibitors to openness amongst team-sport athletes (see Main Theme 3). Indeed, qualitative data from the present study suggested that players are concerned about being perceived by others as ‘weak’ or feel embarrassed about reporting symptoms of concussion while also noting concerns about losing their place in the team. In turn, athletes learn to normalise injury as part of their sporting experience [59,60] and will often hide ‘invisible’ symptoms of concussion to continue playing. Consequently, some sporting bodies might wish to consider investing additional resources and/or making rule changes to include provisions such as concussion spotters and temporary or permanent concussion substitutions that aid accurate concussion identification and diagnosis. Such provisions might also help to alleviate potential ethical dilemmas about reporting symptoms of concussion in teammates, with the emphasis for concussion identification and management on medical personnel and coaches instead (see Main Theme 5). However, such provisions are only ever likely to be introduced at elite levels of sport, meaning researchers and practitioners should continue to explore alternative measures (e.g., developing concussion charters) that might help to address potential inhibitors to openness about concussion amongst amateur players.

When athletes do report symptoms of concussion to others, it is important for teammates and coaches to respond in the right way. Here, qualitative data from the present study highlighted the importance of removing concussed players, having clear plans in place about returning to play, as well as providing reassurance to players (see Main Theme 6). Although concussion management should be a shared responsibility [61] across key stakeholders (e.g., players, medical staff, coaches, referees), knowledge of return-to-play protocols amongst amateur players and coaches is particularly important given that they constitute most of the playing population and medical support is often limited at this level [56]. Without this knowledge, concussions will continue to go unreported and best practice with regards to management will not be implemented [62]. Further, coaches who do not remove players following concussion are neglecting their duty of care to players, as well as signalling to the rest of the team that they do not trust others to play over injured players. Participants also expect coaches to actively support players who report symptoms of concussion, a finding that is corroborated by injury literature [63] on the importance of social support during rehabilitation. Failure to accomplish this will impact the willingness of players to be open about concussions in the future.

In terms of limitations, it is important to note that the present study examined intentions to report symptoms of concussion only. According to the Theory of Reasoned Action and Planned Behaviour, individuals who intend to perform a specific behaviour are more likely to engage in that behaviour [25,26]. However, intentions to report concussive symptoms do not always predict reporting behaviour [21]. Indeed, while championship-level English footballers have moderate concussion knowledge, safe attitudes, and good concussion symptom recognition, many report unsafe concussion behaviours [62]. Thus, future researchers examining concussion reporting intentions should consider the social validity of quantitative measures by examining responses alongside qualitative methods that present an opportunity to identify any contradictions in data. In addition, future researchers should examine the predictive validity of quantitative measures by testing the extent to which responses correlate with desired behaviours, e.g., number of concussions, duration of symptoms, return to play timescales, etc.

A further limitation of this study concerns the generalisability of the sample and analyses undertaken. As such, future researchers may wish to examine transformational leadership and psychological safety as predictors of concussion reporting intentions for athletes in specific sports. There are significant differences in concussion incidence across sports [64] that, in turn, may impact team culture and reporting intentions. Female athletes within sex-comparable sports have also been found to have higher rates of concussion than males [65]. Further, female athletes take longer to become symptom free following sports-related concussion than males [66] and are at an increased risk for developing post-concussive syndrome compared to their male counterparts [67,68]. Although Supplementary File S2 highlights little difference in responses, future researchers may wish to further examine differences in concussion reporting intentions between male and female team-sport athletes [1,69]. In addition, semi-professional athletes have a threefold greater concussion injury rate than amateur athletes and nearly a twofold greater concussion injury rate than junior athletes during match participation, while semi-professional athletes have nearly a 600-fold higher concussion injury risk than professional participants, and nearly a 14-fold higher concussion injury risk than amateur athletes during training participation [70]. Therefore, future researchers may also wish to examine the predictive relationships identified in this study across different levels of competition and at various age groups.

## 5. Conclusions

The present study examined the relationships between transformational leadership, psychological safety, and concussion reporting intentions in team-sport athletes. Quantitative results indicated that transformational leaders supported the development of a psychologically safe environment, within which social norms encouraged team-sport athletes to report their own symptoms of concussion and symptoms of concussion in their teammates. Qualitative findings highlighted the important role that coaches and teammates play in creating a psychologically safe environment. Yet, while education and awareness can be used to further develop such an environment, athletes also identified several barriers to reporting symptoms of concussion. Collectively, these factors impact the willingness of team-sport athletes to be open about symptoms of concussion with others. Thus, interventions that focus on the needs of individual members and encourage regular, open, and honest communication about both sport-specific and personal issues should be encouraged. These interventions should target individual players, teammates, and coaches, with a focus on cultivating open, accepting, empathetic, non-judgemental, and supportive environments, within which discussions about concussion can take place. Interventions should also set clear expectations about what should happen when concussions occur. In so doing, researchers and practitioners may be able to support the development of a team culture that promotes concussion reporting and prioritises player health.

## Figures and Tables

**Table 1:** Main themes, sub-themes, and sample quotes concerning concussion reporting intentions in team-sport athletes.

Main Theme	Sub-Theme	Sample Quotes
(1) Role of Teammates	Be accepting and non-judgemental	‘*… there’s no real, like no judgement among us. Overall, we’re pretty close. We have a laugh, it’s pretty good’.*
		‘*They [their teammates] can pretty much talk to me about anything and I can pretty much talk to them about anything’.*
		*I think it’s about fostering a culture in which they are inclusive of all different people and inclusive of all different kinds of situations, and … the fact that they can come up to me or come up to anyone and speak about their issues.*
	Develop personal relationships	*The majority of us have been together for the last 3 years. 3 or 4 years, so we are quite comfortable with one another, and new girls that come in, we welcome them quite well I feel’.*
		*‘…because of our shared interest and passion for the game, I think we definitely get along…’*
		*‘There is a lot of comradery and a lot of friendships … [and you] … hold each other accountable’.*
		*Constantly talking to them and making sure that they are feeling welcome at every training session at every game. When they come to every training session, we will all go into the changing rooms so making sure I say hi to them each time and asking them how they are going and just acknowledging them all the time.*
		*… lots of conversations were asking me about like you know where I’ve come from, how long I’ve been playing for and just talking about like other interests outside of basketball. I think that also really helps to build relationships.*
		*… after games and training we might have a small snack together or sit outside like in the car park before we go home and talk and just sort of do these things. I think they are really important as it creates that sort of bond away from the pitch.*
(2) Role of Coaches	Encourage accountability	*‘He does that by showing up early to training. Calling upon people to be accountable for themselves’*
		*‘I think the sort of expectation of being there on time and making sure we have the right equipment, the right kit and all that’.*
	Share personal experiences	*… sometimes they would use their own personal experiences, which is motivating for a lot of us because if this person knows what it feels like to be in our position, then they feel like they understand the pressure of games and competitions.*
		*“Now we’re talking about concussion, I’m gonna give you my personal experience of how I got a concussion” and people will start to realise. OK, I haven’t suffered that just on my own. My coach suffered the same thing.*
	Be friendly and approachable	‘*You don’t always want them to be like an authority figure. You need to be able to have some sort of friendly relationship with them, almost like a friend that you can talk to’.*
		*My relationship with them I’d say is really, really good. He is quite laid back I’d say, he is also super approachable. He is super approachable, like legit going up to him and talking to him about how I’m feeling with footie and how I’m feeling off the field … he is very supportive …*
		*He’s been really good with um injuries, like you know, if you have an injury or something is wrong, he’s always been saying, you know, “just tell me, I understand I’ll be here for you”. So even from the start he’s been really open he doesn’t care what it is like he’s like “I don’t care if you broke up with your boyfriend. If you’ve had a really bad day at school or work” like he’s been really good about us trying to make us feel comfortable to go, talk to him.*
	Provide personalised support	*‘… one to one talk with you … like sometimes our coaches call us in like a room and then we have this private talk’*
		‘*… coaches being able to say “you know what you can tell me anything. I’m here to listen, I understand” and that’.*
	Bring the group together	*Partially, like it’s through the coach, sometimes we will do some drills that are more fun … as well as that it requires off the field stuff like sometimes, we might have a day we all just hang out and go to someone’s house, or, we will just have a chat. Or there is, in the warm down, someone might tell a story for example, that happened to them through the week. Erm, you know, it’s building that bit of chemistry off field, erm, or at training…*
(3) Inhibitors and Facilitators to Openness	Hiding symptoms	*‘She did keep a lot of her symptoms a secret from the coach, even though a lot of the girls knew’*
		‘*Sometimes they hide it. Sometimes you see it’.*
	Barriers to reporting	*If a player can get away with playing a game despite putting themselves at risk of being concussed further and injuring themselves further and damaging the brain further, I think a lot of players would take that risk purely for the game time of not being dropped or losing their positions or worrying about someone playing in their position and performing better.*
		*… if you’ve got a mild concussion symptom like maybe it’s just, they’re just feeling a little bit nauseous. Like maybe that could be seen as also a weak thing as well so you can. You should still be able to do this.*
		*Those who haven’t had a concussion don’t understand what the person is going through so I feel like they don’t want to talk about it cause they just feel the other person will not understand.*
	Education and awareness	*Having a coach that has the knowledge about concussions specifically, probably can help prevent more concussions in the future, because that allows them to understand what players are going through as such.*
		*Workshops and lots of discussions about the impacts of it and both long term and short term … It should be done from a grassroots level all the way through the professional athlete programmes just to make sure that people are really aware of the side effects. And you know the difficulties that come with having short-term and long-term concussion.*
		*In Australian Aussie rules, they have just put in a rule that players need to have 12 days off if they’ve been concussed so they can’t play or train during that time. And I felt like because she had just had a concussion and this news … had just come out, I thought it was really poor that she was just able to like to play only like a few days after she did it. And she was feeling like, you know, quite dizzy during those games too.*
		*I think if professional athletes spoke up more it would. Like signal to other athletes like so whatever level you’re playing that, yeah, it’s OK [to talk about concussion].*
(4) Open Team Environment		*I would feel comfortable like being able to talk about it [concussion] because of that comfortable open environment and being able to talk about it with them and like just ‘cause it’s like another injury, they’ll feel like it’s another injury.*
		*… it’s about creating a good culture at the start of the season … and actually addressing the issue. … having a nice overview of the plan just as, not only as a team but also for you individually … If someone does feel the need to come speak out, then they can speak to a couple of people or speak to the coach or erm, you know, if they feel they are at risk of a concussion they can actually come up to the coach and they can feel like, I don’t feel safe playing … It’s about fostering a culture from early on before the season starts, that there is a safe environment where someone can come up and say whatever they please.*
		*… I think we would be quite open about it because we understand that people have their own lives outside of soccer and having a concussion in soccer has to be dealt with in a quite cautious manner. Because if something else goes bad, then their whole life could be impacted.*
(5) Ethical Dilemmas about Reporting Symptoms in Others		‘*… they would certainly talk to me and say I really have this bad headache like I’m starting to feel dizzy … so I will actually inform … coaches to actually do something about it’.*
		*I wouldn’t go up to the coach and say directly what’s wrong because I feel like that would be my teammates responsibility. But I might go up to the coach and say I don’t think so and so is doing so well, he just seems a bit off today or doesn’t seem like himself.*
		*It’s a very sensitive thing for me. Very sensitive because some of them tell it to me and then, they tell me not to tell the coach, not to tell anyone, and if I tell someone that makes me feel like I just betrayed them, so yeah, it’s a very sensitive thing, but I know they need help … I consider my relationship to them and also like my responsibilities.*
		*If their wish was to keep it a bit more private or a bit more reserved, then I would keep it that way … But I think if the problem was getting really bad and was becoming an issue … then I would talk to the coach about it. … I think I would tell the coach only if the person told me to, I’d only ever tell the coach if they couldn’t talk to the coach and really wanted to talk about it. Or erm, I think or, I’d just not talk to the coach at all, and make sure, and constantly check up on the person themselves.*
(6) Response to Reporting	Be supportive and empathetic	*In the past, whenever someone’s been injured or whenever someone’s had a concussion injury, everyone has been super, super supportive. Asking if they are alright or I think with concussion they really know the severity. And concussion is a big factor in Aussie Rules Football. I think the whole game usually stops and especially in a team we are quite caring to the fact we know they can’t come back for the rest of the game and that they will have to deal with those issues. So yeah, we are quite knowledgeable about someone getting a concussion and really just do our best to erm, just get around him and do our best to make sure he feels alright considering.*
		*‘If another one of the players had a concussion before and if the coach is showing that he can support those players, then they’ll feel more comfortable and able to tell [and talk to] the coach’*
		*‘Allowing that coach to talk to the players. And say I understand what you’re going through, we will work through it with you, erm, and you will be back on the pitch in no time’ … and … ‘going to see the player who has just been concussed and encouraging that in the future they will be able to play again.*
	Outline plans for returning to play	*… a plan in place to get you back to playing in a safe manner and also like give you opportunities and you don’t have to feel like that you have to go back early or you have to go back for a period of time just because you have to make sure that you want to keep your spot like I think we need to give like athletes confidence that they’re going to, you know, come back.*
	Remove concussed players	*She just wanted to play … but she wasn’t thinking about the effects of what concussion can actually do to her in the short and the long term. And like the coaches just let it happen even though they knew that she was concussed. So, I think that was a really poor thing by them.*
		‘*That kind of signals to me that the coach doesn’t have as much trust in other point guards in the team’ … and … ‘That was disappointing to see from that aspect of like you know, he’s wanting a concussed player to play instead of like a fully fit player’.*

## Data Availability

The raw data supporting the conclusions of this article will be made available by the authors on request.

## Supplementary Materials

The following supporting information can be downloaded at: https://www.mdpi.com/article/10.3390/ijerph22030393/s1.
