# Supplementary material for: Transformational Leadership, Psychological Safety, and Concussion Reporting Intentions in Team-Sport Athletes
Source: Int J Environ Res Public Health. 2025 Mar 7;22(3):393. doi: 10.3390/ijerph22030393 (PMC11941984; doi:10.3390/ijerph22030393)
Supplement: Supplementary file 1 [file ijerph-22-00393-s001.zip › Supplementary File 1 (Proof Read).pdf]

Supplementary File 1

This supplementary file outlines the processes involved in the development of the Concussion Reporting Intentions Scale (CRIS) used in this study.

Specifically, the CRIS was adapted from the Theory of Reasoned Action and Planned Behaviour questionnaire developed by Register-Mihalik et al. (44). This questionnaire was developed from interviews with high school athletes and focused on the reporting of concussion symptoms. However, the questionnaire developed by Register-Mihalik et al., included variations in the length, tone, and structure of item phrasing, which might impact clarity and coherence. As a result, an iterative process of questionnaire refinement was undertaken, with a focus on item phrasing and language, as well as reviewing the representativeness of items in relation to different sub-scales. The aim here was to develop items that fully capture the individual sub-scales, while minimising the extent to which items tap into concepts other than the one under examination.

Items were extracted from a variety of sources, including reviews of literature, suggestions from experts in concussion, as well as examining existing measures in the area. In instances where the sub-scales were not fully represented, existing items were adapted, or new ones were generated. The wording of all items was discussed and analysed to ensure that item wording was as simple and precise as possible. Here, items were scrutinised for wording and clarity, with ambiguous or unfamiliar language simplified. Item orientation was also considered, as negatively worded items provide a remedial solution to potential response acquiescence.

Following content validity checks, item reduction, pilot testing and initial EFAs, the revised CRIS used in this study included eight items. Two of these items assessed attitudes towards concussion reporting, with higher scores indicating a more favourable attitude towards reporting symptoms of concussion. Two items assessed subjective norms, with higher scores indicating that social referents feel more positive towards the reporting of symptoms of concussion. Two further items assessed perceived behavioural control, with higher scores indicating more feelings of control over concussion symptom reporting, while the final two items assessed intentions to report symptoms of concussion.

When completing the CRIS, participants were asked to consider items in relation to their involvement in sport, as well as their feelings about reporting symptoms of concussion. Participants were also provided with a definition of concussion, against which they could respond to items related to symptom reporting. This definition was the same as that used by Register-Mihalik et al. (21), and stated that: ‘A concussion is an injury caused by a blow to the head or sudden movement of the body followed by a variety of signs and symptoms that may include any of the following: headache, dizziness, loss of balance, blurred vision, ‘seeing stars’, feeling in a fog or slowed down, memory problems, poor concentration, nausea, or throwing up. Getting ‘knocked out’ or being unconscious does *not* always occur with a concussion’.

The first CRIS used in this study examined the willingness of individuals to report their own symptoms of concussion. All items started with the same ‘anchor’, which stated: ‘When I experience possible symptoms of concussion…’. The second CRIS used in this study

examined the willingness of individuals to report possible symptoms of concussion in their teammates. Although the ‘anchor’ was again the same for all items, it was changed to reflect this revised context. Specifically, all items in the second CRIS started with: ‘When I see a teammate displaying possible symptoms of concussion…’. Participants responded to each item on a 7-point Likert scale, ranging from 1 (very strongly disagree) to 7 (very strongly agree). Measures of attitudes, subjective norms, perceived behavioural control, and intentions to report symptoms of concussion were calculated by summing items for each sub-scale.

To examine the factorial validity of the revised CRIS used in this study, two EFAs - with the number of factors to extract fixed to four - were undertaken. The four-factor model that represented the willingness of individuals to report their own symptoms of concussion explained approximately 88 % of the cumulative variance. Table 1 shows the associated variables (items), rotated factor loadings, as well as the Cronbach’s alpha reliability coefficients for each sub-scale (factor). Here, all items loaded onto their hypothesised factors, with no evidence of cross-loading or misloading. In addition, all factor loadings were > 0.71, which is considered excellent. Cronbach’s alpha reliability coefficients for all sub-scales were also > 0.70, indicating adequate internal reliability.

Like the above, the four-factor model that represented the willingness of individuals to report symptoms of concussion in their teammates explained approximately 88 % of the cumulative variance. Table 2 shows the associated variables (items), rotated factor loadings, as well as the Cronbach’s alpha reliability coefficients for each sub-scale (factor). Again, all items loaded onto their hypothesised factors, with no evidence of cross-loading or misloading. All factor loadings were also > 0.71, which is considered excellent. In addition, Cronbach’s alpha reliability coefficients for all sub-scales were > 0.70, which was deemed to be acceptable.

Table 1. Willingness of individuals to report their own symptoms of concussion - EFA factor loadings and Cronbach’s alpha reliability coefficients by sub-scale.

**Factor**	**Associated Variables**	**Rotated Factor Loadings**	**Cronbach’s Alpha Score**
Intentions to Report Symptoms of Concussion	I plan to report them to my coach	0.888	0.871
	I will make every effort to report them to my coach	0.885	
Perceived Behavioural Control	I have no control over reporting them to my coach	0.924	0.867

	I am unable to report them to my coach	0.823	
Attitudes Towards Concussion Reporting	It would be extremely difficult for me to report them to my coach	0.856	0.849
	It would be embarrassing for me to report them to my coach	0.845	
Subjective Norms	People who are important to me would approve of me reporting them to my coach	0.837	0.801
	It is expected of me to report them to my coach	0.829	

Table 2. Willingness of individuals to report symptoms of concussion in their teammates - EFA factor loadings and Cronbach's alpha reliability coefficients by sub-scale.

**Factor**	**Associated Variables**	**Rotated Factor Loadings**	**Cronbach's Alpha Score**
Intentions to Report Symptoms of Concussion	I will make every effort to report them to my coach	0.887	0.875
	I plan to report them to my coach	0.843	
Perceived Behavioural Control	I have no control over reporting them to my coach	0.867	0.863
	I am unable to report them to my coach	0.865	
Attitudes Towards Concussion Reporting	It would be extremely difficult for me to report	0.850	0.865

	them to my coach		
	It would be embarrassing for me to report them to my coach	0.846	
Subjective Norms	It is expected of me to report them to my coach	0.891	0.838
	People who are important to me would approve of me reporting them to my coach	0.745	

In terms of the quantitative measures used in this study, data supported the factorial validity of the CRIS. However, to maximise the utility of these scales, it was decided to focus upon direct measures of attitudes, subjective norms, and perceived behavioural control only. Yet, questionnaires based on the Theory of Reasoned Action and Planned Behaviour often include indirect measures of these constructs as well. In this context, Register-Mihalik et al. (44) defined indirect attitudes as the product of beliefs about concussion reporting and evaluation of these beliefs, indirect subjective norms as a product of normative beliefs (beliefs of important social referents) and motivation to comply with these beliefs, and indirect perceived behavioural control as a product of control beliefs and control belief power (beliefs ‘controlling’ the behaviour and the perceived power of these beliefs). The challenge here then is in finding a balance between questionnaire length and utility. Indeed, the questionnaire used by Register-Mihalik et al. (44) included 48-items, arguably limiting the utility of this measure. On the other hand, the CRIS used in this study included only two items each for attitudes, subjective norms, perceived behavioural control, and intentions, arguably limiting the conceptual coverage of the scales. Thus, we would encourage future researchers to undertake additional psychometric testing using the CRIS developed in this study with a larger sample size.
