# Supplementary material for: Transformational Leadership, Psychological Safety, and Concussion Reporting Intentions in Team-Sport Athletes
Source: Int J Environ Res Public Health. 2025 Mar 7;22(3):393. doi: 10.3390/ijerph22030393 (PMC11941984; doi:10.3390/ijerph22030393)
Supplement: Supplementary file 1 [file ijerph-22-00393-s001.zip › Supplementary File 2 (Proof Read).pdf]

Supplementary File 2

This supplementary file presents the descriptive statistics that consider perceptions of transformational leadership, psychological safety, and concussion reporting intentions for male and female team-sport athletes in this study (see Table 1).

Transformation leadership was assessed using the *Differentiated Transformational Leadership Inventory,* with data from all 27-items collated into one transformational leadership factor, with scores ranging from between 27-135.

Perceptions of psychological safety within a team were assessed using the *Team Psychological Safety* questionnaire. Here, data was collated into one factor, with scores ranging from between 7-49.

The *Concussion Reporting Intentions Scale (CRIS)* assessed attitudes towards concussion reporting, subjective norms, perceived behavioural control, and intentions to report symptoms of concussion. All sub-scale scores ranged from between 2-14.

The first CRIS used in this study examined the willingness of individuals to report their own symptoms of concussion. The second CRIS used in this study examined the willingness of individuals to report possible symptoms of concussion in their teammates.

Table 1. Means (SD) for transformational leadership, psychological safety, and concussion reporting intentions for male and female team-sport athletes.

**Variable**	**Sex**	
	**Male**	**Female**
Transformational Leadership	109.35	112.90
	(16.18)	(16.19)
Psychological Safety	36.27	37.67
	(6.03)	(6.81)
Perceived Behavioural Control	12.24	12.52
(Own Symptoms of Concussion)	(2.18)	(2.50)
Subjective Norms	11.98	12.48
(Own Symptoms of Concussion)	(2.04)	(1.73)
Attitudes	11.78	12.08
(Own Symptoms of Concussion)	(2.47)	(2.39)
Intentions	11.08	11.44

(Own Symptoms of Concussion)	(2.55)	(2.54)
Perceived Behavioural Control	11.09	11.67
(Symptoms of Concussion in Teammates)	(2.67)	(2.35)
Subjective Norms	10.59	11.19
(Symptoms of Concussion in Teammates)	(2.65)	(2.39)
Attitudes	10.92	11.74
(Symptoms of Concussion in Teammates)	(2.69)	(2.53)
Intentions	10.76	11.21
(Symptoms of Concussion in Teammates)	(2.61)	(2.44)
