# Supplementary material for: Transformational Leadership, Psychological Safety, and Concussion Reporting Intentions in Team-Sport Athletes
Source: Int J Environ Res Public Health. 2025 Mar 7;22(3):393. doi: 10.3390/ijerph22030393 (PMC11941984; doi:10.3390/ijerph22030393)
Supplement: Supplementary file 1 [file ijerph-22-00393-s001.zip › Supplementary File 3 (Proof Read).pdf]

Supplementary File 3

This supplementary file includes all analyses of regression, as well as confidence intervals, standardised beta coefficients, significance and R^2 adjusted values from the quantitative analyses in this study.

Here, analyses of regression indicated that perceptions of transformational leadership in coaches significantly predicted perceptions of psychological safety within teams (see Table 1).

Further, perceptions of psychological safety within teams significantly predicted perceived behavioural control (see Table 2), subjective norms (see Table 3), and attitudes towards concussion reporting amongst individuals (see Table 4).

Similarly, analyses of regression indicated that perceptions of psychological safety within teams significantly predicted perceived behavioural control (see Table 5), subjective norms (see Table 6), and attitudes towards reporting symptoms of concussion in teammates (see Table 7).

However, only subjective norms and attitudes significantly predicted intentions of individuals to report their own symptoms of concussion (see Table 8).

In addition, only subjective norms significantly predicted intentions of individuals to report symptoms of concussion in their teammates (see Table 9).

Table 1. Regression analysis summary for transformational leadership predicting psychological safety.

Variable	B	95% CI	β	*t*	*p*
(Constant)	16.596	[11.630, 21.563]		6.584	< 0.001**
Transformational Leadership	0.182	[0.138, 0.227]	0.469	8.064	< 0.001**

Note. R^2 adjusted = 0.216. CI = confidence interval for B. *$p < 0.05$. **$p < 0.001$

Table 2. Regression analysis summary for psychological safety predicting perceived behavioural control concerning the willingness of individuals to report their own symptoms of concussion.

Variable	B	95% CI	β	*t*	*p*
(Constant)	7.224	[5.610, 8.838]		8.819	< 0.001**
Psychological Safety	0.139	[0.096, 0.182]	0.384	6.325	< 0.001**

Note. R^2 adjusted = 0.148. CI = confidence interval for B. *$p < 0.05$. **$p < 0.001$

Table 3. Regression analysis summary for psychological safety predicting subjective norms in relation to the willingness of individuals to report their own symptoms of concussion.

Variable	B	95% CI	β	*t*	*p*
(Constant)	7.819	[6.435, 9.203]		11.133	< 0.001**
Psychological Safety	0.118	[0.080, 0.155]	0.380	6.238	< 0.001**

Note. R^2 adjusted = 0.140. CI = confidence interval for B. *$p < 0.05$. **$p < 0.001$

Table 4. Regression analysis summary for psychological safety predicting attitudes towards concussion reporting amongst individuals.

Variable	B	95% CI	β	*t*	*p*
(Constant)	5.433	[3.764, 7.102]		6.414	< 0.001**
Psychological Safety	0.175	[0.131, 0.220]	0.453	7.712	< 0.001**

Note. R^2 adjusted = 0.201. CI = confidence interval for B. *p < 0.05. **p < 0.001

Table 5. Regression analysis summary for psychological safety predicting perceived behavioural control concerning the willingness of individuals to report symptoms of concussion in teammates.

Variable	B	95% CI	β	*t*	*p*
(Constant)	7.990	[6.061, 9.918]		8.161	< 0.001**
Psychological Safety	0.089	[0.038, 0.141]	0.219	3.405	0.001*

Note. R^2 adjusted = 0.044. CI = confidence interval for B. *p < 0.05. **p < 0.001

Table 6. Regression analysis summary for psychological safety predicting subjective norms in relation to the willingness of individuals to report symptoms of concussion in teammates.

**Variable**	**B**	**95% CI**	**β**	***t***	***p***
(Constant)	8.549	[6.592, 10.507]		8.606	< 0.001**
Psychological Safety	0.061	[0.008, 0.113]	0.148	2.275	0.024*

Note. R^2 adjusted = 0.018. CI = confidence interval for B. *p < 0.05. **p < 0.001

Table 7. Regression analysis summary for psychological safety predicting attitudes towards reporting symptoms of concussion in teammates.

**Variable**	**B**	**95% CI**	**β**	***t***	***p***
(Constant)	5.662	[3.758, 7.565]		5.861	< 0.001**
Psychological Safety	0.150	[0.099, 0.201]	0.356	5.792	< 0.001**

Note. R^2 adjusted = 0.123. CI = confidence interval for B. *p < 0.05. **p < 0.001

Table 8. Regression analysis summary for perceived behavioural control, subjective norms, and attitudes predicting intentions of individuals to report their own symptoms of concussion.

Variable	B	95% CI	β	*t*	*p*
(Constant)	0.934	[-0.926, 2.794]		0.989	0.324
Perceived Behavioural Control	0.045	[-0.099, 0.189]	0.041	0.619	0.536
Subjective Norms	0.652	[0.495, 0.810]	0.501	8.159	< 0.001**
Attitudes	0.150	[0.009, 0.291]	0.144	2.090	0.038*

Note. R^2 adjusted = 0.359. CI = confidence interval for B. *p < 0.05. **p < 0.001

Table 9. Regression analysis summary for perceived behavioural control, subjective norms, and attitudes predicting intentions of individuals to report symptoms of concussion in their teammates.

Variable	B	95% CI	β	*t*	*p*
(Constant)	2.618	[1.361, 3.875]		4.104	< 0.001**
Perceived Behavioural Control	0.061	[-0.063, 0.185]	0.061	0.969	0.334
Subjective Norms	0.584	[0.474, 0.693]	0.590	10.513	< 0.001**
Attitudes	0.117	[-0.007, 0.241]	0.122	1.856	0.065

Note. R^2 adjusted = 0.474. CI = confidence interval for B. *p < 0.05. **p < 0.001
